# Perceptions of prostate cancer patients undergoing definitive radiotherapy on the impact of prostate cancer and radiation therapy on male sexuality

**DOI:** 10.3332/ecancer.2024.1726

**Published:** 2024-07-10

**Authors:** Joseph Daniels, Leroy Baffoe-Krapim, Andrew Yaw Nyantakyi, Edwina Ayaaba Ayabilah, Judith Naa Odey Tackie, Kofi Adesi Kyei

**Affiliations:** 1 National Radiotherapy, Oncology, and Nuclear Medicine Centre, Korle Bu Teaching Hospital, Accra, Ghana; 2 Department of Radiography, University of Ghana, Accra, Ghana; a https://orcid.org/0000-0002-1466-150X; b https://orcid.org/0000-0003-0742-6007; c https://orcid.org/0009-0009-8399-4920; d https://orcid.org/0000-0003-3485-5368

**Keywords:** radiotherapy, prostate cancer, erectile dysfunction, premature ejaculation, sexual function

## Abstract

**Introduction:**

Male sexual function is an important aspect of the life of prostate cancer patients and plays a significant role in the long-term quality of life of prostate cancer survivors. However, there is a paucity of published literature on the perceived impact of prostate cancer and its treatment on the sexual function of patients in Ghana and West Africa in general. The purpose of this study was to explore the perceptions of prostate cancer patients on the effects of the disease and radiation therapy on male sexual function. The study also examined the changes in sexuality experienced by men with prostate cancer.

**Methods:**

This research was a descriptive longitudinal study conducted at the third largest hospital in Africa. The study included Ghanaian prostate cancer patients of all ages who were treated with definitive radiotherapy at the study site between October 2021 and May 2022. Quantitative data were collected and analysed using the Statistical Package for Social Sciences version 26.0 and Microsoft Excel 2019. Descriptive statistics were used to determine frequencies and percentages of the demographic characteristics.

**Results:**

The mean age of the participants was 65.7 years (SD 6.7) ranging from 50 to 81 years. Patients had different ideas about the potential adverse effects of prostate cancer (86%) and radiotherapy (70%) on male sexual function. A decrease in sexual desire (54%) was the commonest perceived effect of prostate cancer on male sexual function followed by premature ejaculation (49%) and a decrease in sexual activity (48%). On the other hand, erectile dysfunction (49%) was the commonest perceived effect of radiotherapy for prostate cancer on male sexual function followed by a decrease in sexual desire (38%) and premature ejaculation (37%). Health professionals were the major source of information regarding the perceptions of the patients on the effects of both prostate cancer (46%) and radiation therapy (43%) on male sexual function.

**Conclusion:**

There should be enhancement of awareness measures to educate Ghanaian cancer patients on the side effects and implications of treatment on their sexuality. Comprehensive sexual health assessment should be incorporated in the routine care of patients with cancers that have the potential to impact the sexual function of patients.

## Introduction

Prostate cancer is the fourth most frequently diagnosed cancer and the eighth leading cause of cancer mortality worldwide [1]. It affects about 60 out of every 100 black men globally [2]. Prostate cancer was the second most frequently diagnosed cancer among males in Ghana in 2020 and the fourth leading cause of cancer-related deaths [1]. The prostate gland is a walnut-sized structure in the male pelvis that surrounds the urethra just below the outlet of the bladder. The gland produces seminal fluid which is a major component of semen. Testosterone is mainly secreted by testicles which control its growth and function. Some men believe developing prostate cancer automatically implies an inevitable impairment of sexual function. However, despite the proximity of the prostate gland to the penis, there is no conclusive evidence that prostate cancer directly causes sexual dysfunction. The sudden reduction in sexual function experienced by some prostate cancer patients has been attributed to psychological instability and depression associated with the diagnosis of cancer [3]. The news of cancer diagnosis is traumatizing and can affect patients emotionally as well as psychologically leading to detrimental changes in their sexuality.

Normal healthy sexual functioning is characterised by the absence of pain or discomfort during sexual activity as well as the absence of physiological difficulty with transitioning through the three-phase sexual response cycle of desire, arousal and orgasm [4]. Additionally, sexual functioning is associated with subjective feelings of satisfaction with the frequency of sexual desire and sexual behaviour, as well as the pleasure derived from engaging in sexual activities. The adverse impacts of various treatment modalities such as surgery, radiotherapy, hormonal therapy and chemotherapy on the male sexual organ can also result in decreased sexual functioning, adversely affecting self-image, perceived identity and the experience of masculinity [5].

Sexuality is an important aspect of life and plays a significant role in the long-term quality of life of cancer survivors [6]. Sexual dysfunction has a significant adversely effect on the quality of life of cancer patients [5]. Various sociocultural factors prevent patients and their care providers from comprehensively discussing the effects of antineoplastic therapy on the sexuality of patients. Even though up to 80% of patients want to learn more about how cancer treatments affect their sexual health and function, about 75% would be hesitant to bring up the topic first [7]. Several studies have been conducted on prostate cancer in Ghana focusing on the risk factors [8, 9], awareness and knowledge [10, 11], prevention [12] and the management of the disease in the Ghanaian setting [13]. However, there is a paucity of published literature on the perceived sexual function of prostate cancer patients in Ghana and West Africa in general.

The purpose of this study was to explore the perceptions of prostate cancer patients about the effects of the disease and radiation therapy on male sexual function. The study also examined the changes in sexuality experienced by men with prostate cancer.

## Methods

### Study design and setting

This research was a descriptive longitudinal study. The study was conducted at the radiotherapy and cancer treatment centre of the third largest hospital in Africa; the Korle Bu Teaching Hospital between October 2021 and May 2022. This facility was established in 1997 and is the largest of three radiotherapy centers in Ghana. The centre operates a 6 MV linear accelerator and a cobalt-60 teletherapy machine and manages patients with solid tumours of all cancer sites. The centre provides cancer care services to Ghanaians and other nationals from several neighboring countries.

### Participants

The study included all Ghanaian prostate cancer patients of all ages who were treated with definitive radiotherapy at the study site between October 2021 and May 2022. The study included eligible patients who had completed their treatment and those who were still receiving radiotherapy during the data collection period for this study. Potentially eligible patients who underwent either adjuvant or salvage radiotherapy were excluded from the study. The purpose of this exclusion was to eliminate the confounding influence of prior surgery (radical prostatectomy) on the sexuality of patients receiving radiotherapy.

### Data sources

A structured questionnaire was used to collect quantitative data from the study participants. The questionnaire elicited information on the changes in sexual function from the time of diagnosis through the commencement of treatment till the end. The questionnaire was formulated on the basis of the International Index of Erectile Function, Prostate Cancer Case Questionnaires, European Organisation for Research and Treatment of Cancer (EORTC) Quality of Life Questionnaire – Prostate Cancer Module (EORTC QLQ-PR25), University of California, Los Angeles (UCLA) Prostate Cancer Index (UCLA-PCI) and the Expanded Prostate Cancer Index Composite Sexual Assessment and Erectile Dysfunction (ED) Questionnaire. The questionnaires were modified to include items that elicited information on patients’ perception of the implication of the diagnosis of prostate cancer and radiation treatment on their sexual function. Clinical information was extracted from the electronic medical records of the patients. The items in the questionnaire were independently reviewed by two experienced radiotherapists and one radiation oncologist to ensure that the questions were well formulated to elicit the required information. The reliability of the research instrument was tested with Cronbach’s alpha.

### Study size

A purposive sampling method was used to select patients for the study. The appropriate sample size for the study was determined to be 60 patients based on Slovin’s formula [14]. The sample size included all consenting prostate cancer patients undergoing definitive radiotherapy for localised disease.

### Data management and statistical methods

The data collected on patients were stored and processed on a secure computer. The quantitative data collected were analysed using the Statistical Package for Social Sciences version 26.0 and Microsoft Excel 2019. Descriptive statistics were used to determine frequencies and percentages of the demographic characteristics.

### Ethical considerations

Ethical approval was obtained from the institutional review board prior to the commencement of this study. All patients recruited into the study provided prior written informed consent. The information collected on the patients were used solely for the purpose of this study. The data collected were anonymised to remove all patient-identifying information to ensure the privacy of patients. The confidentiality of the patients and their medical data were always ensured during the conduct of this study.

## Results

### Baseline characteristics of the study participants

In all, 98 Ghanaian patients with prostate cancer were screened for potential eligibility to participate in the study. A total of 75 patients were found to be eligible, out of which 63 (84%) provided written informed consent and participated in the study. The mean age of the participants was 65.7 years (SD 6.7) ranging from 50 to 81 years. In all, 41 participants (65%) were married whereas 11 (17.5%) were single or divorced (Table 1).

Table 2 summarizes the clinical and treatment-related characteristics of the study participants. In all, 36.5% (*n* = 23) had International Society of Urological Pathology (ISUP) grade group 1 tumour which corresponds to a Gleason score of 3 + 3. Also, 11 (17.5%) and 17 (27%) had tumours categorised as ISUP grade groups 2 and 3, respectively. Most of the patients has high-risk disease whereas 8 (12.7%) had very high-risk disease. There were no patients with very-low-risk disease. The commonest dose of radiotherapy prescribed was 78 Gy delivered in 39 fractions. Most of the patients (*n* = 49, 77.8%) were treated with intensity-modulated radiotherapy (IMRT).

### Perception of the effects of prostate cancer on male sexual function

**Figure 1.**
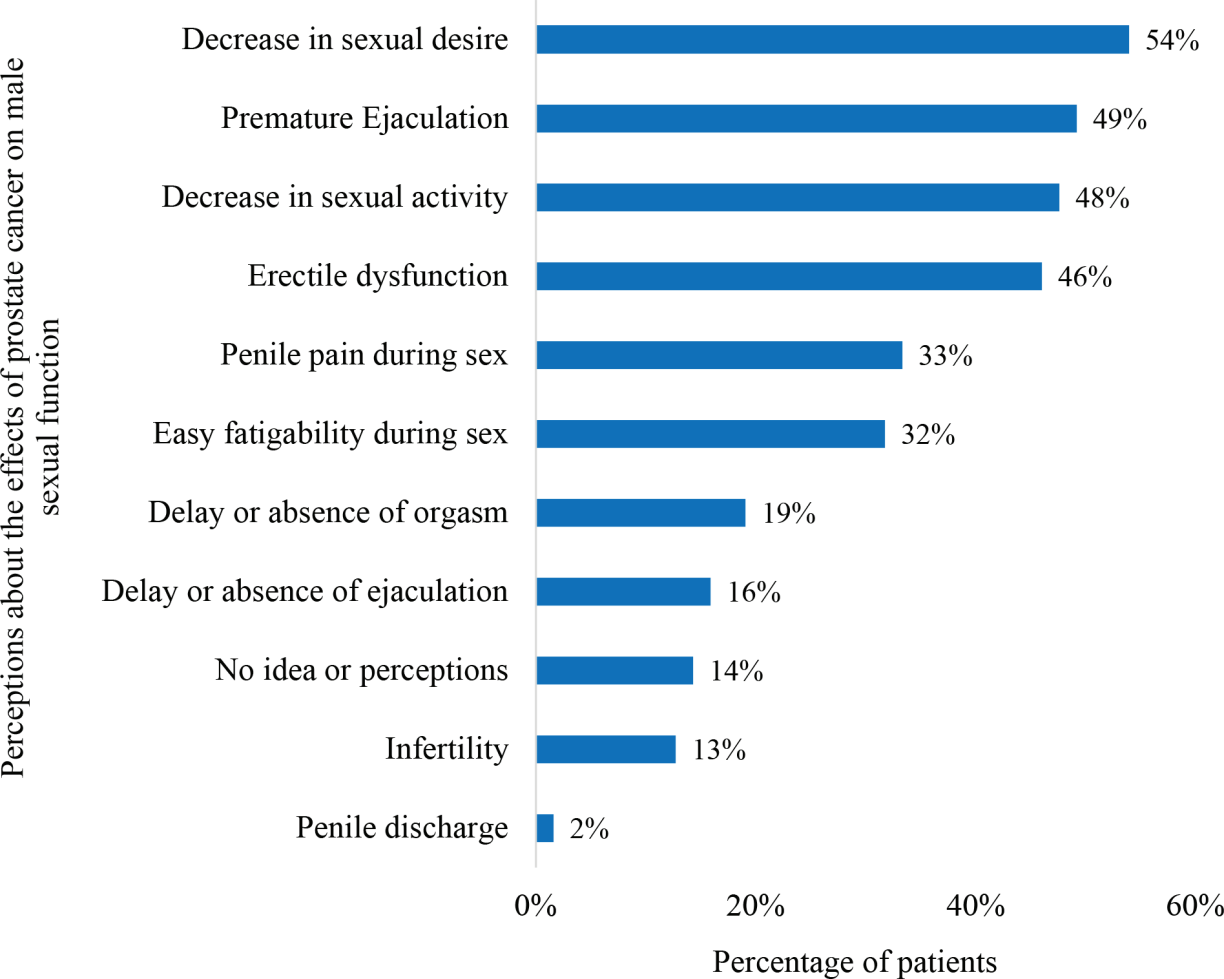
Patients’ perceptions of the effects of prostate cancer on male sexual function.

Figure 1 summarizes the perceptions of the study participants on the effects of prostate cancer on male sexuality. In all, 86% of participants had different kinds of perceptions whereas 14% had no idea or perceptions on how prostate cancer affects the sexuality of men diagnosed with the disease. The commonest perceptions were ‘decrease in sexual desire’ (54%), ‘premature ejaculation’ (49%), ‘decrease in sexual activity’ (48%) and ‘ED’ (46%). Eight patients (13%) had the perception that prostate cancer is associated with male infertility.

### Perception of the effects of radiation therapy for prostate cancer on male sexual function

**Figure 2.**
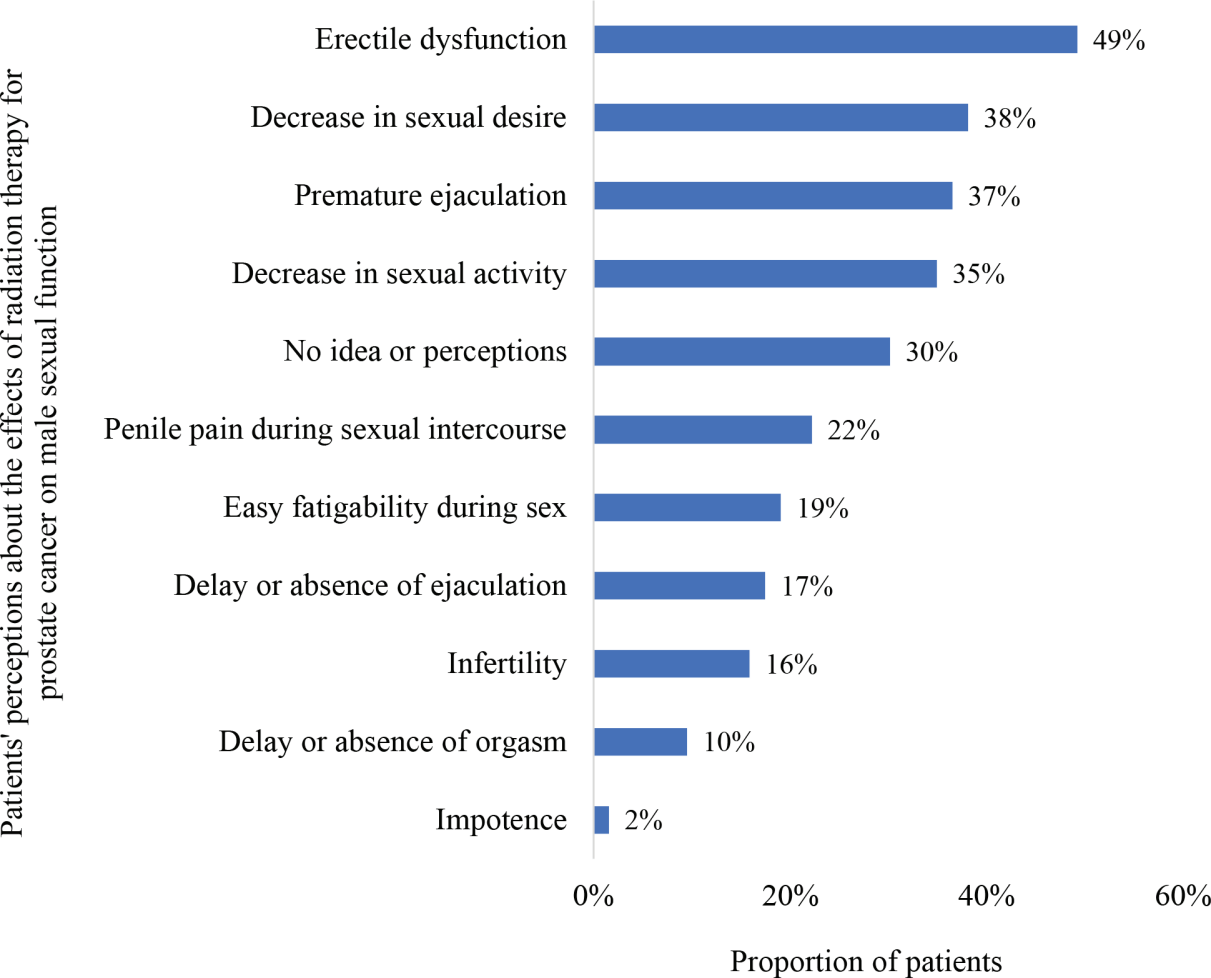
Patients’ perceptions of the effects of radiation therapy on male sexual function.

Figure 2 illustrates the different perceptions held by the study participants on the effects of radiation therapy for prostate cancer on male sexual function. Overall, 70% of the patients held different opinions whereas 30% had no idea or perceptions about the effects of radiotherapy on male sexual function. The most common perceptions were ‘ED’ (49%), ‘decease in sexual desire’ (38%), ‘premature ejaculation’ (37%) and ‘decrease in sexual activity’ (35%). Only 16% and 2% had perceptions of infertility and impotence, respectively, as effects of radiotherapy on male sexual function.

### Sources of information regarding patients’ perceptions on the effects of prostate cancer and radiation therapy on male sexual function

**Figure 3.**
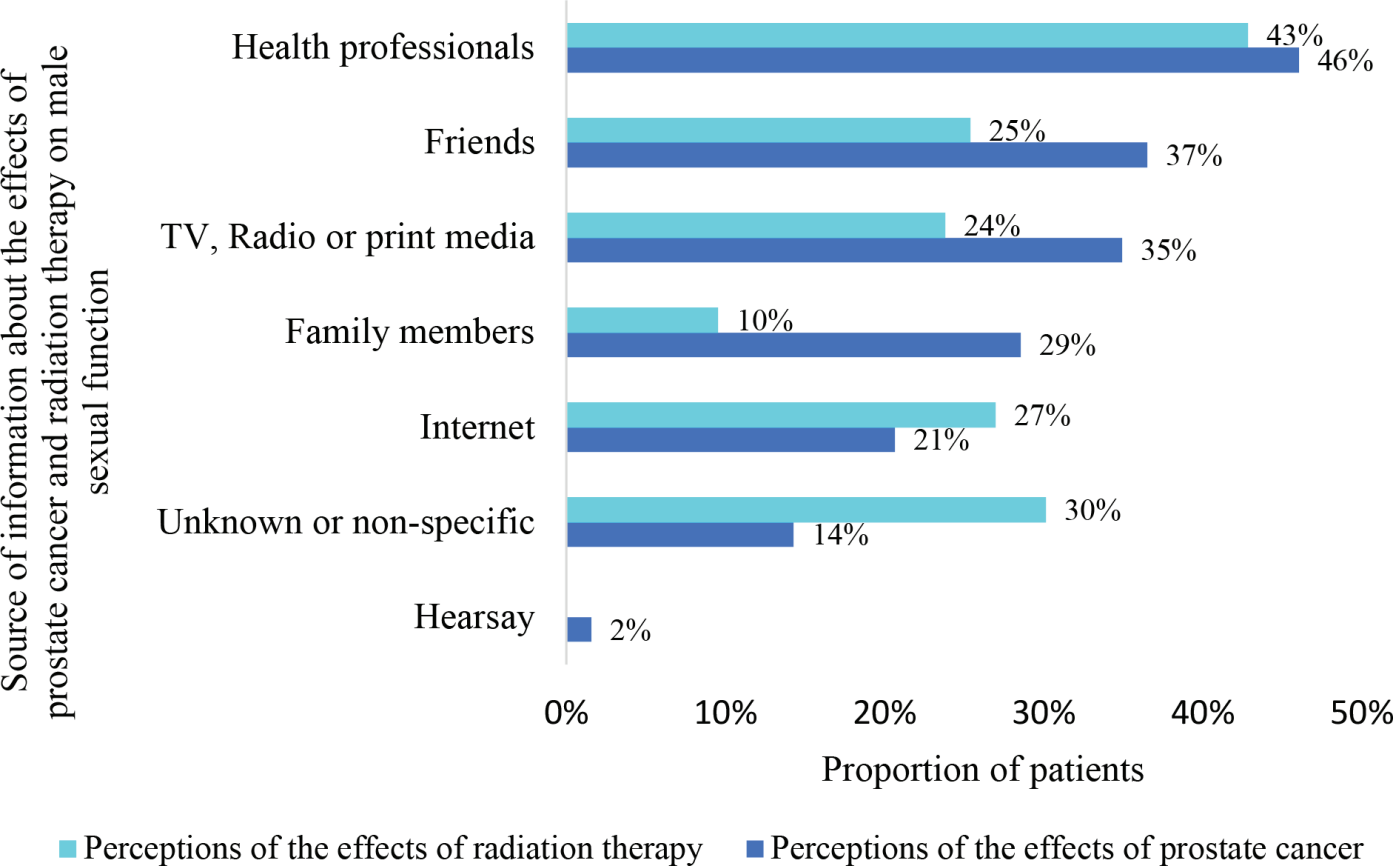
Sources of information on effects of prostate cancer and radiation therapy on male sexual function.

Health professionals were the major source of information regarding the perceptions of the participants on the effects of both prostate cancer (46%) and radiation therapy (43%) on male sexual function. In all, 25%, 24% and 10% obtained information about the effects of radiation therapy from friends, television (and radio or print media) and family members, respectively. On the other hand, many more patients received information on the effects of prostate cancer on male sexuality from friends (37%), television (and radio or print media) (37%) and family members (29%). The internet was the source of information for 21% and 27% regarding the effects of prostate cancer and radiation therapy, respectively, as depicted in Figure 3.

### Changes in sexual function among prostate cancer patients undergoing radiation therapy

Table 3 summarizes the changes in sexual function experienced by patients with prostate cancer undergoing radiation therapy. In all, 35 participants (56%) were still sexually active whereas 28 (44%) no longer engaged in sexual activity (intercourse). In all, 47 patients (75%) were dissatisfied with their overall sexual function, 24 (38%) had lost confidence for sexual intimacy, whereas 47 (75%) had experienced an overall deterioration of their sexual function.

### Prostate cancer patients’ perception of their body image

The perception of the study participants on their body image is summarized in Table 4. In all, 23 patients (37%) had never experienced anxiety whereas the rest had experienced anxiety either always (2%), often (93%), sometimes (25%) or rarely (33%). Additionally, 34 (54%) were always happy with themselves and 21 (33%) always felt full of life. Furthermore, 33(52%) always felt calm and peaceful whereas 2(3%) always felt either downhearted and/or depressed. Only 9 participants (14%) never had the feeling of helplessness or hopelessness.

## Discussion

The mean age of the study participants was 65.7 years (SD 6.7) ranging from 50 to 81 years. This is similar to a documented mean age of 65 years among men diagnosed with prostate cancer [15]. A study describing the sexual function of 50 patients with prostate cancer treated with radiotherapy for localised disease also reported a mean age of 68.8 years [16].

A high proportion of patients had one idea or another, about the potential adverse effects of prostate cancer (86%) and radiotherapy (70%) on male sexual function. This indirectly suggests that a significant proportion of the patients diagnosed with prostate cancer had some basic knowledge and familiarity with the disease. This observation can be attributed to the widespread ongoing educational and awareness creation campaigns regarding the prevention, detection and management of prostate cancer in Ghana. However, the proportion of prostate cancer patients who had no idea about the potential effects of the disease (14%) and its treatment (30%) on male sexual function is worrisome. It points to a gap in patient education especially at the time of diagnosis and during treatment. All cancer patients are required to provide documented informed consent prior to treatment with radiation therapy. A crucial aspect of the process of obtaining informed consent for treatment is the discussion of potential side effects. Healthcare providers must strive to ensure that all patients are given adequate information in a well-understood language of the patient’s choice about their diagnosis and potential side effects of the recommended treatment prior to the commencement of therapy.

Health professionals were the major source of information regarding the perceptions of the patients on the effects of both prostate cancer (46%) and radiation therapy (43%) on male sexual function. This finding highlights the important role of health workers in the dissemination of accurate and reliable information to prostate cancer patients. For health workers to fulfill this role effectively, they must be adequately educated in the prevention and screening of cancers in general as well as relevant diagnostic modalities and treatment options. The internet was the source of information for 21% and 27% regarding the effects of prostate cancer and radiation therapy, respectively. There is no doubt that the internet plays a major role in the dissemination of information regarding both malignant and nonmalignant diseases. A study published in 2021 revealed a disturbing trend of health content and communication on social media, particularly regarding cancer and chronic diseases like diabetes mellitus [17]. It behooves on editors of social media platforms and publishers of cancer blogs and websites to ensure that scientifically accurate information is made available to readers; therefore, such articles should be verified by competent healthcare providers in the field before publication.

In all, 25%, 24% and 10% obtained information about the effects of radiation therapy from friends, television (and radio or print media) and family members, respectively. On the other hand, the perceptions of patients on the effect of prostate cancer on male sexuality were formed based on information obtained from friends (37%), television (and radio or print media) (37%) and family members (29%). This trend of information acquisition indirectly attests to the fact that the print media as well as friends and family members of prostate cancer patients tend to know more about prostate cancer than radiotherapy. Stakeholders in cancer education must expand the reach of educational campaigns to include close relations and family members of individuals diagnosed with cancer. The role, usefulness and impact of radiotherapy must also be integrated into cancer awareness creation campaigns to dispel any myths that might exist regarding the use of radiotherapy in the management of cancer patients.

Prostate cancer and its treatment can have significant adverse impacts on male sexuality [18]. These impacts often extend beyond physical changes in the body or treatment-related side effects. Impaired sexual function is a well-recognised adverse effect of prostate cancer treatment irrespective of the modality of treatment be it radiotherapy, surgery, chemotherapy or androgen deprivation therapy (ADT) [19]. Significant contributing factors to the impairment of sexual function among prostate cancer patients include psychological distress, anxiety, depression and relationship strain [20]. Hence, the importance of addressing these aspects alongside antineoplastic therapy cannot be overemphasised in the effective management of prostate cancer patients. In this study, 57 prostate cancer patients (90%) reported a decrease in sexual activity whereas 47 (75%) reported deterioration and dissatisfaction with their sexual function. This trend is consistent with the findings of a study that explored the Scandinavian prostate cancer patients’ sexual problems and satisfaction with their sex life following antineoplastic therapy [21]. They reported a low level of sexual satisfaction among prostate cancer patients who were sexually active at the time of diagnosis and attributed this to a high prevalence of sexual health problems. Another study also reported the occurrence of decreased libido, ED and altered orgasmic function among many survivors of prostate cancer [22, 23].

A decrease in sexual desire (54%) was the commonest perceived effect of prostate cancer on male sexual function followed by premature ejaculation (49%), decrease in sexual activity (48%) and ED (46%). About 35%–60% of prostate cancer patients are estimated to experience radiotherapy-related ED which often leads to severe distress among these patients [24, 25]. In this study, ED (49%) was the commonest perceived adverse effect of radiotherapy for prostate cancer on male sexual function followed by decrease in sexual desire (38%), premature ejaculation (37%) and decrease in sexual activity (35%). ED refers to the inability to achieve or maintain an erection of the male sexual organ long enough to have penetrative sexual intercourse. It has been identified as an important priority for men with localised prostate cancer when selecting suitable treatment modalities [26]. ED has been reported among men with prostate cancer who are treated with radiotherapy or radical prostatectomy as well as those who do not receive active treatment [27].

In all, 35 participants (56%) were still sexually active whereas 28 (44%) were no longer engaged in sexual activity (intercourse). A study observed that sexual intimacy was completely out of the question for some men with prostate cancer [28]. Men with prostate cancer refrain from sexual activity due to several reasons including fear of infection, fatigue, the perception of adverse impact of sexual activity on the outcome of their treatment and the fear of exposing their sexual partners to radiation [16]. Orgasmic functions, erection function and patients’ general satisfaction with their sexual lives have been reported to diminish with radiation treatment [16]. The quality of life of prostate cancer patients is adversely affected by adverse changes in their sexuality that occur during or because of treatment [29].

Prostate cancer diagnosis and treatment has an adverse impact on patients' physical and emotional well-being [30]. A study on body image, self-esteem and sense of masculinity in patients with prostate cancer also surmised that, the side effects of treatment and impacts of the diagnosis of prostate cancer itself may be responsible for the depression that the affected patients undergo [31]. In this study, only 14 patients (22%) felt depressed always, often or sometimes. Most of the patients 45 (71%) felt full of life either often or always whereas 51 (81%) often or always felt calm and peaceful. The majority of the patients were also generally happy with themselves and rarely (33%) or never (37%) felt anxious. This may be attributed to the strong patient support network that exists at the study site for patients with solid tumours of all body sites including the prostate. Such vital networks are essential in helping prostate cancer patients to adopt effective coping mechanisms to deal with the impairment of their sexual function.

## Recommendations

There should be enhancement of awareness-creation measures to educate Ghanaian cancer patients on prostate cancer diagnosis and the side effects/implications of treatment for their sexuality. Comprehensive sexual health assessment should be incorporated in the routine care of patients with cancers that have the potential to adversely impact the sexual function of patients.

## Limitations

The use of ADT in the management of prostate cancer patients is associated with sexuality-related side effects. Some of the patients in this study had concomitant ADT with radiotherapy however the study did not separately explore the contribution of ADT alone to the impairment of sexual function among the patients.

## Conclusion

This study provides valuable insights into the diverse perceptions of prostate cancer patients regarding the impact of prostate cancer and its definitive radiotherapy treatment on male sexuality. The findings highlight that ED is perceived as the most prevalent effect of radiotherapy on male sexual function. This perception is notably influenced by information disseminated by healthcare professionals, underscoring the critical role of medical guidance in shaping patient expectations and experiences. Understanding these perceptions is crucial for healthcare providers to address concerns effectively, offer appropriate support and enhance the quality of life for patients facing the dual challenges of prostate cancer and its treatment. Future research should continue to explore these dynamics to further refine patient education and support mechanisms, ensuring that patient care is both holistic and responsive to the nuanced impacts of cancer treatments on sexual health.

## Conflicts of interest

The authors declare no competing interest.

## Funding

This study did not receive any specific funding support from funding agencies in the public, commercial, or not-for-profit sectors.

## Author contributions

JD: Conceptualisation, Methodology, Validation, Supervision, Visualisation, Writing – Original Draft, Writing – Review and Editing LB-K: Conceptualisation, Methodology, Formal analysis, Investigation, Writing – Original Draft AYN: Writing – Review and Editing EAA: Writing – Review and Editing JNOT: Writing – Review and Editing KAK: Writing – Review and Editing, Supervision.

## Data availability

The data used to support the findings of this study are available from the corresponding author upon reasonable request.

## Figures and Tables

**Table 1.** Baseline characteristics of the study participants (*n* = 63).

Variables	Number	Percentage (%)
Age		
< 60 years	13	20.6
60–70 years	33	52.4
> 70 years	17	26.2
Marital status		
Single	11	17.5%
Married	41	65%
Divorced	11	17.5%

**Table 2.** Clinical and treatment-related characteristics of the study participants (*n* = 63).

Variables	Number	Percentage (%)
Clinical stage		
T1C	10	15.9
T2A	5	7.9
T2B	12	19
T2C	27	42.9
T3A	5	7.9
T3B	4	6.4
ISUP grade group		
1	23	36.5
2	11	17.5
3	17	27
4	12	19
5	0	0
Risk group		
Very low risk	0	0
Low risk	6	9.5
Favourable intermediate risk	4	6.3
Unfavourable intermediate risk	10	15.9
High risk	35	55.6
Very high risk	8	12.7
Prescribed dose		
78 Gy in 39 fractions	46	73.0
74 Gy in 37 fractions	7	11.1
60 Gy in 20 fractions	10	15.9
Radiotherapy technique		
3D-CRT	14	22.2
IMRT	49	77.8

ISUP: International Society of Urological Pathology, IMRT: intensity modulated radiotherapy

**Table 3.** Prostate cancer patients’ personal experience of changes in sexual function.

Change in sexual function	Present		Absent		Not applicable *N*/(%)
	*N*	%	*N*	%	
Sexual activity	35	56	28	44	-
Impotence	6	10	57	90	-
Delay or absence of orgasm	15	24	20	32	28 (44)
Absence of ejaculation	10	16	25	40	28 (44)
Premature ejaculation	7	11	28	44	28 (44)
Easy fatigability during sex	32	51	31	49	28 (44)
Penile pain during sexual intercourse	5	8	30	48	28 (44)
Decrease in sexual activity	57	90	6	10	-
Decrease in sexual desire	25	40	38	60	-
ED	20	32	43	68	-
Overall deterioration of sexual function	47	75	16	25	-
Loss of confidence for sexual intimacy	24	38	39	62	-
Dissatisfaction with overall sexual function	47	75	16	25	-

**Table 4.** Patients’ perception of their body image.

Questionnaire items	Responses *N* (%)				
	Aways	Often	Sometimes	Rarely	Never
Do you feel full of life ?	21 (33)	24 (38)	15 (24)	2 (3)	1 (2)
Do you feel anxious ?	1 (2)	2 (3)	16 (25)	21 (33)	23 (37)
Do you feel nothing could cheer you up ?	4 (6)	1 (2)	4 (6)	25 (40)	29 (46)
Do you feel calm and peaceful ?	33 (52)	18 (29)	9 (14)	2 (3)	1 (2)
Do you feel downhearted and depressed ?	2 (3)	2 (3)	10 (16)	20 (32)	29 (46)
Are you happy with yourself ?	34 (54)	20 (31)	7 (11)	1 (2)	1 (2)
Do you feel hopeless or helpless ?	1 (2)	14 (22)	23 (37)	16 (25)	9 (14)
